# Communication during telemedicine consultations in general practice: perspectives from general practitioners and their patients

**DOI:** 10.1186/s12875-024-02576-1

**Published:** 2024-09-04

**Authors:** Amy D Nguyen, Sarah J. White, Tim Tse, John A. Cartmill, Peter Roger, Sarah Hatem, Simon M. Willcock

**Affiliations:** 1 Centre for Health Systems and Safety Research, Australian Institute of Health Innovation, Macquarie University, Sydney, NSW 2109 Australia; 2 St Vincent’s Clinical Campus, UNSW Sydney, Sydney, NSW Australia; 3 Centre for Social Impact, UNSW Sydney, Kensington, NSW Australia; 4 Macquarie Medical School, Faculty of Medicine, Health and Human Sciences, Macquarie University, Sydney, NSW Australia; 5 Department of Primary Care, Faculty of Medicine, Health and Human Sciences, Macquarie University, Sydney, NSW Australia; 6 Department of Linguistics, Faculty of Medicine, Health and Human Sciences, Macquarie University, Sydney, NSW Australia

**Keywords:** Telemedicine, Telehealth, General practice, Communication, Primary care

## Abstract

**Background:**

Telemedicine allows delivery of healthcare to occur between parties that are not in the same location. As telemedicine users are not co-present, effective communication methods are crucial to the delivery and reception of information. The aim of this study was to explore perspectives of general practitioners (GPs) and patients on the interactional components of telemedicine consultations.

**Methods:**

Semi-structured qualitative interviews were held with telemedicine users; 15 GPs and nine patients self-selected from a larger telemedicine study. Participants were asked about their preparation for telemedicine consultations, conducting telemedicine consultations and post-consultation activities. Deidentified transcripts from the interviews were analysed thematically.

**Results:**

GPs and patients discussed factors they used to decide whether a consultation would be best conducted by telemedicine or in-person; the condition to be discussed, the existing doctor-patient relationship and whether physical examination was required. Participants also described how they prepared for their telemedicine consultations, gathering relevant documents, and reading previous notes. Participants described strategies they employed to optimise the telemedicine interaction; improving conversational flow and building rapport, as well as difficulties they experienced when trying to provide and receive care via telemedicine.

**Conclusions:**

Patient factors including health literacy and familiarity with technology affect the transfer of information shared during telemedicine consultations and consideration of these factors when choosing patients for telemedicine is required. Many GPs and patients have innate communication skills to effectively deliver and receive care through telemedicine. However, they may not be aware of these subconscious techniques to use to optimise telemedicine consultations. Communication training could be delivered to increase conversational flow, build rapport, and establish safety netting.

**Supplementary Information:**

The online version contains supplementary material available at 10.1186/s12875-024-02576-1.

## Introduction

Telemedicine encompasses the use of telecommunications technologies, such as telephone and videoconferencing, to deliver healthcare services and has transformed healthcare delivery, enabling remote consultations, monitoring, and treatment [1]. In Australia, like internationally, the COVID-19 pandemic significantly accelerated the adoption of telemedicine, particularly within general practitioner (GP) clinics, serving as a critical tool to ensure continuity of care while minimising the risk of virus transmission [2]. The transition to telemedicine has presented challenges in effective communication [2]. Currently, there is a lack of comprehensive support and guidance in this area [2, 3]. Clinicians have primarily relied on trial and error and ad hoc web resources to navigate telemedicine interactions [4, 5]. The shift from in-person consultations to telephone or video calls for delivery of clinical care can have a substantial impact on patient satisfaction and clinical outcomes, as it alters relationship-building and other communication practices, such as non-verbal cues, physical examinations and establishing rapport and trust [2, 6]. 

One of the fundamental differences between in-person consultations and telemedicine lies in the reduction or absence of visual cues. This altered communication landscape poses unique challenges that impact the quality of the interaction and thus patient outcomes. Nonverbal cues, such as facial expressions and body language, are considered important to building rapport and establishing mutual understanding. These can be downgraded, lost, or compromised in telemedicine, [7] which may affect patient engagement, empathy, and information exchange [8]. 

Existing guidelines and checklists exist for the technical aspects and privacy dimensions of telemedicine. These include the selection of appropriate technology platforms and data security, but there is a noticeable lack globally, of specific recommendations for communication between GPs and patients during telemedicine consultations [2, 3]. 

This study explores the perspectives of Australian GPs and their patients using qualitative interviews focusing on the communication and interactional components of telemedicine consultations.

## Methods

This research is part of a larger study informing the development of guidelines to optimise communication and enhance patient-centered care in the telemedicine landscape [2]. 

### Recruitment and participants

Data collected between February to November 2022, occurred initially across two general practice clinics in Sydney, Australia. To increase the sample size, further GPs with experience of telemedicine were recruited through an expression of interest email using the professional networks of TT and SMW and a snowballing technique [9]. However, additional GPs did not have to work at the two general practice clinics. Patients who had experienced telemedicine within the last twelve months at the two general practice clinics were emailed a survey regarding their telehealth experiences, which allowed them to select if they would be open to be contacted for an interview [2]. Patients who indicated interest were contacted using telephone and email by researchers for an interview. Informed consent was obtained from all participants prior to starting the interview.

### Data collection

An open-ended interview guide, developed by the research team based on previous telemedicine research and literature, was used to support the semi-structured interviews, which allowed participants to direct the interview within their experiences of telemedicine (Supplementary Material A, GPs; Supplementary Material B, Patients). Interview topics included preparing for telemedicine consultations, the conduct of the consultations as well as post-consultation activities, and were briefly described to participants prior to the start of the interview. During interviews, telemedicine referred to both participants’ use of telephone and videoconferencing. Semi-structured interviews were conducted over the phone by one of two researchers who were experienced in conducting qualitative interviews in a health context (AN (PhD), SH (MPH)). Interviewers and participants were not known to each other prior to the interviews. All interviews were audio recorded and transcribed by a professional transcription service (Pacific Transcription) using ‘intelligent verbatim’. Transcripts were de-identified to ensure participant confidentiality. Transcripts were not cross checked by participants for comment.

### Analysis

Transcriptions were thoroughly examined by two researchers (SH, AN) using thematic analysis in NVivo 12 [10]. During this process, all transcripts were read independently by the two researchers to identify initial codes arising from the discussions with interview participants. Researchers then met to discuss and organise these codes into themes and subthemes. These themes and subthemes were then developed into an analysis framework using consensus to establish the predominant themes and subthemes relevant to the research questions. This analysis framework was then used to reanalyse all transcripts to ensure it encapsulated all themes. Variations between reviewers were resolved by consensus. A Consolidated criteria for reporting qualitative research (COREQ) checklist is included as Supplementary Material C.

### Ethics

Ethics for this research was granted by Macquarie University Human Research Ethics Committee HREC EXEC Medical Sciences Committee, Application #9534.

## Results

Thematic saturation was reached following a total of 24 interviews (15 interviews with GPs (average duration = 27.9 min (range = 14–48 min) and nine interviews with telemedicine patients (average duration = 13.2 min (range = 5–49 min)).

The 15 GPs and nine patients that participated discussed many aspects of telemedicine (both telephone and videoconferencing modalities) during the interviews. They discussed the factors that impacted on their selection of modality for their consultations i.e., either telemedicine or in-person, how they prepared for their telemedicine consultations, their communication during the telemedicine consultations and difficulties they faced using telemedicine.

### Contextual factors of telemedicine consultations

Several reasons for choosing a telemedicine consultation over an in-person appointment were articulated by GPs and patients. Conditions that were perceived as easier to deal with due to their simpler or routine nature, as opposed to those requiring physical examinations, were considered most suitable for telemedicine. Patients often preferred telemedicine over in-person consultations for convenience, especially if they lived rurally, were elderly or had young children.

*“Pure convenience*,* because my GP… can be 45 minutes late even if you’re the first appointment of her morning… That’s not a problem when you’re at home. You just have your phone near you*,* you keep going about your business. You put the washing on*,* you do some ironing*,* you do your other emails*,* doesn’t matter when she calls really.” (Patient #1)*.

GPs also expressed reasons related to convenience when they were screening a patient for preferred consultation mode. Some patients also said that if the call was for a sensitive matter that might upset them, they preferred telemedicine so that they could react in a location of their own choosing.

*“I actually prefer to do these in a safe space*,* so that I don’t*,* you know*,* walk out crying… I much prefer telehealth for those conversations.” (Patient #4)*.

GPs noted that bad news was not delivered by telemedicine, with GPs preferring patients to come in if that was the case.

*“I try not to deliver bad news via telemedicine. I guess you lack the facial expression and the connection as if you were going to do it face-to-face.” (GP #2)*.

GPs stated that they used telemedicine as a triage service at times, where they would attempt to solve the issue remotely in the first instance, and then only if they were not able to, they would get the patient to come in.

*“In most cases we would have a teleconsult first as a triage call. If we can solve the problem during this call*,* then great. If not*,* then by the end of their history*,* we tend to be able to formulate a fairly good idea of whether or not this patient needs to come in and we would arrange for the consultations accordingly.” (GP #3)*.

An issue that frequently arose in interviews with both patients and GPs was the concept of ‘safety netting’, although individual participants used a range of different terms when discussing it, rather than explicitly calling it safety netting. Safety netting (which encompasses the more familiar notion of ‘duty of care’) occurs when doctors and patients mutually agree on the level of care that can be satisfactorily delivered within a particular setting (in this case, via a telemedicine or an in-person consultation). Both groups of interviewees stated that the decision was largely dependent on the purpose of the consultation. If the reason for the consultation was simple, such as getting a prescription or referral, both GPs and patients agreed that telemedicine was acceptable.

*“If I was only needing a script or if I was only needing a referral… I would think that’s [telemedicine] a very good way… All I need to say*,* it’s my time to see my specialist and my referral is out of date*,* could I please have another referral?” (Patient #9)*.

When asked about the nature of telemedicine consultations, both GPs and patients stated that consultations often focused on a single topic. This was due to the relatively short duration of a standard telemedicine consultation (as per telemedicine funding models in Australia), but also related to the fact that telemedicine consultations were often scheduled for less complex issues.

*“I find that most of the consultations - both over the phone and online - have mostly been single topic issues. I think patients have been generally good in understanding that that sort of medium is not necessarily the best for talking and bringing multiple issues to the doctor. That’s probably something best left to an extended face-to-face consultation.” (GP #11)*.

It was reported by GPs that there were groups of patients that exhibited resistance to telemedicine, particularly the elderly or those less familiar with technology, which limited opportunities for telemedicine.

### Conducting a telemedicine consultation

GP participants were asked how they went about conducting telemedicine consultations. Asked about training, many participants stated that they had received no or limited training in telemedicine. Those GPs that did receive training said that the training was primarily focused on how to use telemedicine technology and did not extend to ways of optimising communication with patients who were not in the same room.

Before beginning a telemedicine consult, GPs had to confirm they were speaking to the correct patient. GPs reported asking identity questions, such as asking for their date of birth, as well as recognising the patient’s voice, if they were a regular patient.

GPs stated that most of their telemedicine consultations were via telephone as opposed to videoconferencing, this being due to patient preference, convenience, or technical issues. Patients also said they preferred telephone over videoconferencing; for some, this was due to previous negative experiences with video and that the telephone is a more familiar mode of communication than video.

*“I tend not to be camera ready all the time*,* so I’m not super in love with the video.” (Patient #1)*.

*“Initially I try [to connect via videoconference]*,* sometimes it doesn’t*,* sometimes it does and sometimes I just log in so that she knows I’m waiting*,* but the videos are terrible*,* and I just don’t even use it.” (Patient #4)*.

Funding models in Australia at the time of the study for GPs to conduct a telemedicine consultation required that they must have seen the patient in person within the last 12 months, [11] hence most of their telemedicine patients were not new to them. However, some participants noted that when a patient’s usual GP was not available, this patient then had to see another GP within the same practice. Both GPs and patients commented that this was not their preference.

*“The only difficulties we’ve had is more if we’ve had to pick a different GP because [usual doctor] is not available and then trying to communicate that has been a bit harder*,* but [new doctor] has been really good at dumbing it down to the right level and then answering all the questions and giving us time to ask questions if we weren’t sure on like the action plan or something like that.” (Patient #7)*.

In these instances where GPs were seeing a patient new to them, GPs had to ensure that they shared the notes from the telemedicine consultation with the patient’s usual GP to support continuity of care as much as possible. It should be noted that sharing of patient notes occurs in Australian general practice clinics through patients having a central record that can be accessed by all GPs working in that clinic.

*“I also try and not overstep the boundary because if someone has a regular doctor*,* I generally a lot of the time say look*,* you can follow up with your regular doctor. I’ll make sure that the notes are clear so they know what’s going on when you see them again. If there’s anything important*,* I’ll send them a message or talk to them*,* give a formal handover.” (GP #12)*.

GPs expressed concern with treating new patients via telemedicine because of issues with continuity of care and that they needed more information from the patient leading to a longer appointment.

*“It’s [new patient] more difficult because you don’t know the patient as well*,* you haven’t got that initial information or relationship with the patient. Depending on what the problem was… that certainly takes much longer with new patients.” (GP #14)*.

When GPs were asked about ending telemedicine consultations, they noted that it was important to end the call by ensuring the patient understood what they were being told. GPs reported that they employed several methods to ensure this, such as asking patients to repeat information or asking questions to “quiz” the patient.

*“I’ll often say*,* hey*,* because I can’t write this down*,* can you just repeat back to me what I think’s happening? And I’ll say*,* do you need to write that down*,* and then we might together make another appointment… So*,* I’ll just clarify the follow-up that I’m expecting.” (GP #15)*.

GPs also reported that they provided opportunities for patients to ask final questions at the end of their telemedicine consultations to ensure their understanding.

*“Definitely giving them an opportunity at the very end of the consultation to ask any questions or anything else they wanted to bring up. I will do that for patients both in person and in the telemedicine consultation as well. But I might ask that a little bit more over the phone*,* just because I can’t tell if the - for the body language*,* if there’s something else they wanted to talk about. I do do that throughout the consultation*,* particularly at the end.” (GP #8)*.

Regarding follow-up post-telemedicine consultation, such as requiring a prescription or referral to be sent to the patient, administration staff would generally be responsible for this. However, GPs seemed to be more likely to book patient follow-ups during telemedicine consultations, especially when the patient was to come back in-person or if it was urgent.

### Communication during a telemedicine consultationn

Communication was impacted during telemedicine given limited, if any, visual support, facial expressions, and body language. GPs and patients faced additional difficulties including the obvious inability to conduct physical examinations. Patients expressed concerns regarding GPs’ communication when using jargon and the fact that patients required adequate health literacy to understand concepts remotely.

*“For telemedicine*,* I don’t know how you’d do it without sounding discriminatory*,* but it’s almost like how confident they [patients] are speaking English and understanding. A doctor can wind down their vocabulary*,* but… that vocabulary is quite expanded… So not so much big words*,* but unusual words for someone who’s learned English as a second language.” (Patient #1)*.

During telemedicine consultations, GPs said that they were also left wondering at times whether patients truly understood what they were being told as they could not see their facial expressions.

*“I actually feel that I’m more keen to clarify with a telemedicine consultation than a face to face. It may actually be a positive bias… The fact that you don’t see them might clarify them more. Whereas I think you take for granted with the non-verbal cues that they’ve been looking at you*,* they’ve looked receptive… I think it’s [telemedicine consultations] actually better for me because I always go look*,* are you happy with that plan? Do you have any questions? So what I want you to do is this and if this happens do this. I generally clarify. I find that I clarify the plan a bit better to be honest*,* because you don’t have that extra cue. Whereas if someone is sitting there and they’re nodding… I probably feel less inclination for clarification to be honest.” (GP #12)*.

GPs reported employing several mitigation strategies to deal with the challenges of imparting complex information when providing remote care. These strategies included screen sharing, directing patients to a particular website, and even accompanying the patient to the website.

*“Sometimes I actually go*,* ‘Are you in front of a computer?’*,* and people usually are*,* and I run through a website and talk about it together… I’ll say google this image and then I’ll talk to them about it. Google this page and then I’ll run them through it*,* so I do use that as a technique… I direct them to a very specific resource and go through something together so that they have something to continue to read after. Rather than go have a look at this website when you get home*,* I just get them to bring it up and I talk to them about it*,* go through it with them.” (GP #14)*.

Multitasking can affect effective conversational flow [12]. Multitasking occurred in telemedicine consultations from both the GP and the patient. Tasks completed by GPs while they were on telemedicine consultations included gathering of extra information for the consultation such as looking up patient notes, information about conditions being discussed and searching for advice to provide to patients. GPs also took patient notes during their telemedicine consultations. While GPs were self-aware of their own multitasking, they noted that these additional tasks were not any different to what they would conduct in in-person consultations.

*“If the patient is talking*,* I’ll be writing notes*,* or if I already have a plan that I want them to XYZ blood tests*,* I’ll start to kind of get that going on the computer. I usually tend to find it’s easier to multitask on a phone consult*,* because then I can put the patients on speaker*,* or through a headset*,* then I’d have my hands free to do all the paperwork stuff*,* like ordering tests*,* in-between writing notes or writing referral letters. It’s probably a little bit easier to do that over the phone than face-to-face because when you’re face-to-face you tend to want to engage with the patient*,* you have eye contact – you don’t want to be looking at the screen the whole time. Whereas if you’re on the phone it doesn’t really matter*,* you can just keep typing away and doing other things*,* like actioning the tasks of the consult.” (GP #11)*.

However, reassurance, in the form of the GP narrating their actions as they were performing tasks, was reportedly used to signal to the patient that they were still on the phone and actively listening.

*“Perhaps sometimes there’s long pauses if I’m looking up something and need to concentrate. But I might say something like*,* I just need to look this up*,* do you mind holding on a couple of minutes. So*,* I guess long pauses are me*,* but I try and tell the patient*,* try and explain to the patient what I’m doing.” (GP #6)*.

Interestingly, some of the patient participants did not at first realise that they conducted any form of multi-tasking during telemedicine consultations until prompted. GPs noted tasks that patients carried out while on remote consultations included driving, going shopping, and tending to children or pets.

*“Some people are taking this call – they’re in the car or in the park or somewhere else and then there’s kind of distractions or dropping out and that could be very off putting in terms of flow for you as the doctor*,* depending on what they’re thinking. If they are in their home and then if they get disturbed by other things*,* other people or whatever else is going on in their home*,* that can break the flow.” (GP #9)*.

GPs and patients agreed that relationship building was key. This was no different when patients were conducting telemedicine consultations with a GP that was not their usual doctor. GPs and patients reported that they used a variety of methods to quickly build a relationship with one another remotely. GPs built rapport and assessed patient understanding by adjusting the way they spoke such as using a friendly voice and speaking slower, while listening to the others’ speaking tone.

*“Obviously*,* the non-verbal cues are sort of out the window*,* because you’re not visually seeing anything. It’s more*,* I guess*,* the nature of what you say and how you say it. I think from our dynamics today*,* you probably get a bit of a feel that I have probably a rather friendly voice and I guess the way that I’m speaking to you today is probably how I would speak to my patients as well. So*,* it’s using effective communication. Try and assess understanding*,* through clarification and just being just generally friendly*,* you know?” (GP #1)*.

Other conversational flow strategies said to be employed during telemedicine by both interviewee groups included providing each other space to speak and leaving long pauses to minimise overlapping speech.

*“With the telephone consults*,* I mean obviously what’s lacking there is the facial expressions. I think leaving gaps and giving the patient the opportunity to speak a little bit more*,* rather than interrupting them prematurely. I think it’s much easier in person because you can see the visual cues there if there’s something else they want to say. But obviously I can’t tell over the phone. So*,* I think just leaving a little bit more of a gap in terms of silences*,* at least in the beginning of the consultation to let the patient speak.” (GP #8)*.

*“We just have a natural flow of conversation*,* so it sort of works. I mean obviously I listen to [doctor] and wait until she finishes.” (Patient #4)*.

Further conversational flow strategies such as small talk and asking questions, were also said to be used by both GPs and patients to improve communication over the phone, with GPs using information found in the patient’s notes as prompts.

*“If there is something documented I will bring it up. I will be like oh*,* you just came back from travel last year*,* how was it*,* or oh*,* the doctor has started you on these new meds*,* how’s it going? Rather than just going how is everything*,* what can I do for you*,* like I will bring those things up so there’s some sort of illusion of familiarity. That’s one of the techniques I frequently use to gain rapport with patients.” (GP #12)*.

## Discussion

Broadly speaking, both GP and patient participants saw telemedicine as a convenient consultation method for simple indications where no physical examinations are needed. Additionally, telemedicine facilitated safety netting between GPs and patients, through remote triaging to minimise unnecessary in-person presentations, whilst allowing patients to receive adequate and timely care, and provide understanding of when they need to follow-up. Communication strategies reportedly used by GPs during telemedicine included a running commentary when they were engaged in an otherwise solo activity such as looking up a result. A deliberate warm and friendly voice, and even encouraging small talk were also reported as proactive strategies employed by GPs. Conversational flow was facilitated by ensuring adequate space for each other to speak. Appropriately timed questions confirming understanding were also effective in telemedicine consultations.

### Practical implications

When planning improvements to telemedicine, the findings of this study suggest that digital literacy and health literacy should both be considered when deciding when and how telemedicine is used, given that patients had concerns regarding use of medical terminology and GPs noted that those with less digital experience tended to show some resistance to using telemedicine; areas of concern that have been demonstrated in literature [13, 14]. This extends to considerations of how GPs and patients might be supported through training and upskilling. GPs need to consider a patient’s digital literacy, often related to their age. GPs should be mindful of these considerations when deciding whether telemedicine is the appropriate method of delivery of care. Health literacy is an important factor in health care. This is particularly important as in telemedicine, the patient and doctor often cannot see each other’s expressions and therefore comprehension of information cannot be easily verified [15]. For example, the facial twinge that can indicate doubt or uncertainty to a skilled communicator is missing or attenuated during telemedicine [16]. In such cases, there is a risk that explanations from either party simply plough on with the speaker being unaware that some clarification is necessary.

This study demonstrated that GPs were conscious of implementing alternate ways of ensuring understanding, particularly important towards the end of calls, such as sharing links to websites, screen-sharing to show important information, asking patients questions to confirm understanding, and asking patients to write things down. It should be noted that some of these methods will be less effective for those who are not digitally literate, as screen-sharing and shared viewing of websites will be difficult or impossible in such circumstances. This shows that GPs need to be creative as they relay complex information during telemedicine, because drawing diagrams, physical demonstrations, and gestures are often not available. Additionally, it has been reported that clinicians require even higher-level communication skills when conducting consultations remotely [17]. Research has shown that patients can interpret information from telephone consultations differently than intended [18]. From a patient’s perspective, they can support their own understanding by asking questions and repeating information back to the GP in their own words, but this requires agency. Patient agency is facilitated when the GP and patient have a good relationship, as with good rapport, patients are likely to feel more comfortable about participating proactively in the consultation [19]. However, this can be undermined when there is an imbalance in authority, which has been reported in telemedicine consultations being more clinician-centred, as opposed to controlled by the patient [20]. 

Several observations that were discussed in interviews were heightened when a GP sees a patient new to them. For example, when the patient is new to the doctor, information collection must be deliberate and comprehensive because of the risk of false hypothesis and confirmation bias [21]. Even if the consultation is a one-off, the GP needs to ensure that they take detailed notes to share with the patient’s regular doctor to support continuity of care [22, 23]. This may mean that a longer consultation is required in order for the patient to be able to describe their relevant medical and social history to the GP, and so indication and patient familiarity to the doctor should be screened at the point of booking a telemedicine appointment [15]. This process may require changes to funding models for adequate GP reimbursement for longer appointments.

As telemedicine becomes an established part of the mainstream healthcare landscape, there are several issues that the overall health sector needs to address [24]. Given that the COVID-19 pandemic which necessitated the increased use of telemedicine was recent, the majority of GPs currently working were educated and trained when in-person consultations were the focus, with little to no training provided for telemedicine. This study showed that how to conduct a telemedicine consultation has not been adequately taught to clinicians; instead, GPs have been thrown into it and expected to adapt, an observation also demonstrated in literature [25]. Therefore, a large gap exists in the training of doctors to effectively deliver care to patients remotely [26, 27]. For those already in clinical practice, evidence-based continuing medical education workshops focusing on the strategic adjustments necessary to conducting safe and effective telemedicine consultations would no doubt be welcomed, with changes to the curriculum in the educating and training of doctors also required. Theory-informed reflective ‘teach-back’ methods, encompassing training workshops and ongoing self-reflection, have been used in telemedicine settings successfully previously [28]. Therefore, the older models of teaching communication skills at medical schools need to be reviewed to include the full spectrum of communication formats that are in use currently, such as through telemedicine [29]. 

From a government perspective, technical difficulties experienced by participants in this study, but also published elsewhere, demonstrate that telemedicine infrastructure, such as internet connection, telephone reception and telemedicine devices, needs improvement [30, 31]. Further, certain populations should be targeted to upskill for effective telemedicine use, such as those who are elderly or digitally illiterate [32]. In these populations, a focus on telephone only rather than videoconferencing may be more effective as telephone is more straightforward and familiar.

Future research should explore the practical implications arising from this study and evaluate their effectiveness in practice. Further, important implications for telemedicine future research include consideration of the role of a person’s family or nominated carer in supporting their use of telemedicine. This is particularly important for people who have limited access to healthcare or higher demands for healthcare, such as those living in remote locations or who require support in their activities of daily living. It would also be beneficial to explore which health events trigger the need to use telemedicine, and how the use of telemedicine changes accordingly.

### Strengths and limitations

A limitation of the study was that no demographic data was collected from participants. This meant that we could not stratify the findings according to characteristics such as participants’ age, rurality and experience with telemedicine, as well as potentially limiting the generalisability of the findings. All patients were recruited from two clinics, which could affect their perspectives, e.g. access to telemedicine. However, given that patients can have telemedicine experiences with multiple healthcare providers (e.g. GPs, specialists, allied health professionals), the specific context of each participant is less relevant to this study as the patient participants were asked to discuss their overall impressions of telemedicine, not just those provided by the GP they had most recently seen. Similarly, GPs can practice at multiple clinics and therefore their telemedicine experiences shared in this study may not be specific to just one clinic, providing more robust findings. A strength of the study was that recruitment and data collection occurred until thematic saturation occurred. This ensured that more representative perspectives of telemedicine were collected.

## Conclusions

Even with these sector-wide considerations, telemedicine offers great opportunities for convenient and effective healthcare. The learnings from the forced use of telemedicine during the COVID-19 pandemic, where both clinicians and patients discovered that they could communicate effectively using technology, show that the use of telemedicine is likely to remain but may still be in its fundamental stages. As its use increases, there is an opportunity to optimise telemedicine with formal training in the techniques identified in this study. By following the communication principles garnered from this study, including rapport building, attention to conversational flow and facilitation of safety netting, general practice telemedicine consultations can be optimised for effectiveness and patient safety.

## Electronic supplementary material

Below is the link to the electronic supplementary material.

Supplementary Material 1

Supplementary Material 2

## Data Availability

The datasets used and/or analysed during the current study available from the corresponding author on reasonable request.
