# Supplementary material for: Communication during telemedicine consultations in general practice: perspectives from general practitioners and their patients
Source: BMC Prim Care. 2024 Sep 4;25:324. doi: 10.1186/s12875-024-02576-1 (PMC11373490; doi:10.1186/s12875-024-02576-1)
Supplement: Supplementary file 1 — Supplementary Material 1 [file 12875_2024_2576_MOESM1_ESM.docx]

# Supplementary Material A – Interview Guide (General Practitioners)

1. What training or support have you been provided for telemedicine i.e. both telemedicine and video conferencing?
2. What resources do you access before telemedicine consultations?
3. How do your telemedicine consultations differ between new and regular patients?
4. How do you determine whether a consultation can be via telemedicine as opposed to F2F?
   1. Are there conditions that you think are better suited to telemedicine than others?
5. What proportion of your telemedicine consultations are via phone vs videoconferencing?
6. How do you confirm you are speaking to the correct patient?
7. What methods do you use to build rapport with patients given you cannot see them?
8. Do you multitask during telemedicine consultations?
9. How often are your patients multitasking during a call?
10. Are your calls generally regarding one topic?
11. How do you deliver bad news during telemedicine?
12. What difficulties have you faced regarding:
13. Flow of conversation
14. Difficult topics to discuss
15. No visual cues from patient
16. Limited physical examinations
17. Technical issues
18. Patient resistance to telemedicine
19. What methods do you use to ensure patients have understood what you have told them?
20. How do you provide opportunities for patients to ask further questions?
21. What methods do you use to organise follow-ups such as additional tests or appointments the patient needs to undergo?

# Supplementary Material B – Interview Guide (Patients)

1. Why do you book a telemedicine consultation (i.e. telemedicine and videoconferencing) over face-to-face?
2. Do you prefer your telemedicine consultations to be via telephone only or with video as well?
3. Do you use a mobile device, landline or computer to receive telemedicine?
4. What information or documents do you prepare before your telemedicine consultations?
5. Do you multitask during telemedicine consultations?
6. Are your calls generally regarding one topic?
7. What difficulties have you faced regarding:
8. Flow of conversation
9. Difficult topics to discuss
10. Rapport building
11. No visual cues from doctor
12. Limited physical examinations
13. Technical issues
14. Doctor resistance to telemedicine
15. What issues have you had with understanding the information doctors provide to you during telemedicine consultations?
    1. What do you do if you do not understand something?
16. Do you feel that your doctor provides opportunities for you to ask further questions?
17. Do you ever follow-up with your doctor after telemedicine consultations?
